# Phase Modulators Based on High Mobility Ambipolar ReSe_2_ Field-Effect Transistors

**DOI:** 10.1038/s41598-018-30969-7

**Published:** 2018-08-24

**Authors:** Nihar R. Pradhan, Carlos Garcia, Bridget Isenberg, Daniel Rhodes, Simin Feng, Shahriar Memaran, Yan Xin, Amber McCreary, Angela R. Hight Walker, Aldo Raeliarijaona, Humberto Terrones, Mauricio Terrones, Stephen McGill, Luis Balicas

**Affiliations:** 1 Department of Chemistry, Physics and Atmospheric Sciences, Jackson State University, Jackson, MS 39217 USA; 2 National High Magnetic Field Laboratory, Florida State University, Tallahassee, FL 32310 USA; 3 Department of Physics, Florida State University, Tallahassee, FL 32306 USA; 4 Lincoln High School, Tallahassee, FL 32311 USA; 5 Department of Physics and Center for 2-Dimensional and Layered Materials, The Pennsylvania State University, University Park, PA 16802 USA; 6 Engineering Physics Division, Physical Measurement Laboratory, NIST, Gaithersburg, Maryland 20899 USA; 7 Rensselaer Polytechnic Institute, Department of Physics, Applied Physics, and Astronomy, Troy, NY 12180 USA; 8 Department of Materials Science & Engineering, The Pennsylvania State University, University Park, PA 16802 USA; 9 Department of Chemistry, The Pennsylvania State University, University Park, PA 16802 USA; 10 Institute of Carbon Science and Technology, Faculty of Engineering, Shinshu University, Nagano, 380-8553 Japan

## Abstract

We fabricated ambipolar field-effect transistors (FETs) from multi-layered triclinic ReSe_2_, mechanically exfoliated onto a SiO_2_ layer grown on *p*-doped Si. In contrast to previous reports on thin layers (~2 to 3 layers), we extract field-effect carrier mobilities in excess of 10^2^ cm^2^/Vs at room temperature in crystals with nearly ~10 atomic layers. These thicker FETs also show nearly zero threshold gate voltage for conduction and high ON to OFF current ratios when compared to the FETs built from thinner layers. We also demonstrate that it is possible to utilize this ambipolarity to fabricate logical elements or digital synthesizers. For instance, we demonstrate that one can produce simple, gate-voltage tunable phase modulators with the ability to shift the phase of the input signal by either 90° or nearly 180°. Given that it is possible to engineer these same elements with improved architectures, for example on *h*-BN in order to decrease the threshold gate voltage and increase the carrier mobilities, it is possible to improve their characteristics in order to engineer ultra-thin layered logic elements based on ReSe_2_.

## Introduction

Layered rhenium-based transition metal dichalcogenides (TMDCs), or ReX_2_ where X = S, Se) are the subject of a renewed interest due to their unique anisotropic optoelectronic properties^1–6^. Due to a lattice distortion these materials crystallize in a distorted triclinic-1*T*′-phase instead of the more conventional trigonal prismatic, or 2*H*-phase, or the rhombohedral or 3*R*-phase. The crystal structure of ReX_2_ is special due to their in-plane motif, i.e. four Re atoms are arranged in a diamond-like shape with these diamonds forming atomic chains along the *b*-direction^7^.

In contrast to other more intensively studied layered dichalcogenides such as Mo(S,Se)_2_ or W(S,Se)_2_, which display a transition from an indirect to a direct band gap when exfoliated down to the monolayer limit^8^, the Re-based TMDCs show nearly layer-independent (ReS_2_)^9^, or very weakly layer-dependent (ReSe_2_), optical and vibrational properties^6^. This has been interpreted as evidence for an extremely weak inter-planar coupling although angle resolved photoemission spectroscopy observes an out-of-plane electronic dispersion, indicating that in fact the interlayer coupling in ReSe_2_ is appreciable^10^.

However, their triclinic symmetry makes both compounds optically biaxial, resulting in an anisotropic planar response with respect to the optical polarization^3,11,12^. The Raman-active modes of thin layers of both compounds have also been found to be anisotropic^13–19^.

According to Density Functional Theory (DFT) calculations, bulk and monolayer ReS_2_ have nearly identical band structures with direct bandgaps of 1.35 eV and 1.44 eV, respectively^2^. These values are relatively close to those extracted from high resolution electron energy loss spectroscopy, which finds direct band gaps of 1.42 eV and of 1.52 eV for bulk and monolayer ReS_2_, respectively^17^. In contrast, the DFT calculations in ref.^19^ indicate that ReS_2_ displays an indirect band gap that is very close in energy with respect to the direct one in the bulk and also in the monolayers. Photoemission in ref.^10^ suggests that ReSe_2_ would also possess an indirect band gap at all thicknesses which is consistent with Local Density Approximation calculations revealing gaps of 0.92 eV for the bulk and 1.22 eV for monolayer^3^. However, photoluminescence measurements at *T* = 10 K coupled to GW and Bethe-Salpeter calculations find that the bandgap of ReSe_2_ increases as the number of layers decreases, displaying values of 1.37 eV for the bulk and of 1.50 eV for the monolayer, while maintaining a direct band gap and independence of the number of layers^3^. In comparison with the other TMDCs crystallizing in 2*H*-phase, a direct band gap approaching ~1.5 eV would make these compounds particularly appealing for photo-sensing and photovoltaic applications (according to the Shockley-Queisser limit). However, as seen from the above results, the nature of the band gap in ReSe_2_, i.e. direct or indirect, remains unclear. To date, there are no reports on room temperature PL from ReSe_2_. Despite our multiple attempts, we were also unable to collect room temperature PL data from this compound, although we can easily extract a PL signal from those compounds crystallizing in the 2 *H* structure, which are known to display an indirect band bap in the bulk^8^. This observation is particularly difficult to reconcile with a direct band gap.

Nevertheless, photodetectors^20^ based on high-quality chemical vapor deposition grown ReS_2_ were reported to yield photoresponsivities as high as *R* $\cong$ 604 A/W corresponding to an enormous external quantum efficiency EQE = 1.50 × 10^5^% and a specific detectivity^21^ D^*^ $\cong$ 4.44 × 10^8^ m (Hz)^1/2^/W. For ReS_2_ stacked onto *h*-BN, values as high as *R* = 88 600 A/W, EQE = 2 × 10^7^%, and D^*^ = 1.182 × 10^10^ m (Hz)^1/2^/W were reported^22^, indicating that the substrates play a significant role on the performance of these compounds. As for ReSe_2_, values as high as *R* = 3.68 × 10^4^ A/W were reported after improving the resistance of the source contact via a triphenyl phosphine-based *n*-doping technique^23^. These pronounced photoresponsivities would suggest that the band gap of these materials might indeed be direct.

Here, we show that FETs based on multilayered triclinic ReSe_2_ mechanically exfoliated onto SiO_2_, display ambipolar behavior with very small threshold back-gate voltages, current ON to OFF ratios exceeding 10^6^ and electron field-effect mobilities approaching 380 cm^2^/Vs at room temperature. We find that Density Functional Theory calculations can replicate the observed Raman spectra for the bulk and for the monolayers concluding that all active Raman modes belong to the *A*_g_ irreducible representation given that inversion is the only symmetry operation compatible with its structure. We also show that the ambipolarity of ReSe_2_ opens up interesting opportunities for complementary logic electronics: for instance, we demonstrate that it is easy to produce very simple, gate-voltage controlled AC-voltage phase modulators as previously reported for ReS_2_^24^.

## Results and Discussion

**Figure 1:**
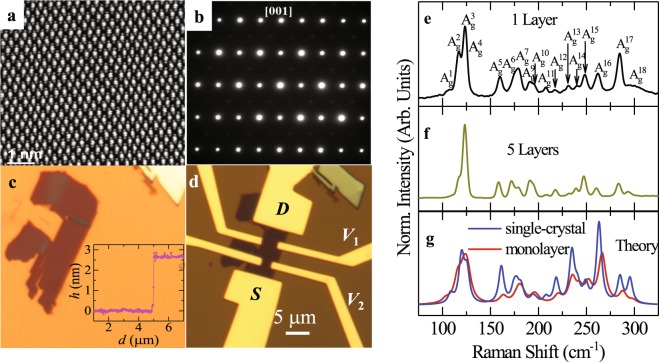
(**a)** Scanning transmission electron microscopy (STEM) image of an exfoliated ReSe_2_ single-crystal displaying a chain-like atomic arrangement. (**b**) Electron diffraction pattern for a ReSe_2_ single-crystal when the incident electron-beam is perpendicular to the planar atomic arrangement showing a rectangular planar Brillouin zone. (**c**) Micrograph of a typical few layered ReSe_2_ single-crystal exfoliated onto SiO_2_ which from AFM (inset) had a step height of 2.7 nm, or was four layers thick (for an inter-planar lattice separation *c* = 0.6702 nm^5^). (**d**) Micrograph of the same crystal after deposition of the electrical contacts which consisted of 50 nm of Au on a 5 nm layer of Cr. The larger electrical contacts were used to source (S) and drain (D) the current *I*_ds_ when performing two-terminal measurements. The smaller contacts V_1_ and V_2_ were used for voltage sensing in four-terminal measurements. For this sample, the separation between the current leads was *L* $\cong$ 10.5 μm, the width of the channel was *w* $\cong$ 3.6 μm and the separation between voltage leads was *l* $\cong$ 4.5 μm. (**e**) Raman spectra of a ReSe_2_ monolayer. Given that inversion symmetry was the only symmetry operation present in the monolayer, in few-layers and in the bulk, there was just one Raman active irreducible representation, i.e. *A*_g_. Therefore, all peaks were associated with Raman *A*_g_ modes. (**f**) Raman spectra for a crystal composed of five atomic layers. (**g**) Theoretical Raman spectra for monolayer (red) and bulk (blue) ReSe_2_.

Figure 1a displays a scanning transmission electron microscopy (STEM) image of one of our exfoliated ReSe_2_ single-crystals. The high crystallinity of these crystals is confirmed by the electron diffraction pattern collected along a direction perpendicular to the planes or along the [001] direction (see Fig. 1b). Given its structural symmetry and similarity to ReS_2_, ReSe_2_ also tends to exfoliate in the form of nearly rectangular flakes^18,19^, as seen in Fig. 1c, which shows a micro-image of the ReSe_2_ flake exfoliated onto a 285 nm thick SiO_2_ layer grown on *p*-doped Si. As shown in the inset, according to atomic force microscopy (AFM) the thickness of the exfoliated crystal shown in 1c is approximately four atomic layers for an inter-planar lattice separation *c* = 0.6702 nm^5^. Figure 1c shows a micro-image of the ReSe_2_ flake exfoliated onto a 285 nm thick SiO_2_ layer grown on *p*-doped Si. Figure 1d shows the same crystal after the deposition of the electrical contacts, i.e. 50 nm of Au on 5 nm of Cr. The electrical contacts were deposited using standard e-beam lithography and e-beam evaporation techniques. This configuration of six contacts allows one to measure the Hall-effect to extract the Hall-mobilities which will be reported elsewhere. Figures 1e,f display the experimental Raman scattering spectra for a monolayer and a five layer crystal, respectively. Their expected theoretical spectra, from which we index the peaks in Fig. 1e, are shown in Fig. 1g. In order to compare with the experimental Raman results, ab-initio density functional theory (DFT) and density functional perturbation theory (DFPT) calculations were performed for monolayer and bulk ReSe_2_ as implemented in the plane wave code CASTEP^25,26^. The starting structure for the bulk crystal was obtained from Lamfers *et al*.^27^. Monolayer, few-layer and the bulk crystal exhibit just inversion symmetry and belong to the ${\rm{P}}\overline{{\rm{1}}}$ space group. Local density approximation (LDA) using the Ceperly-Alder-Perdew and Zunger (CA-PZ) functional^28,29^ with 6 × 6 × 1 Monkhorst-Pack *K*-points and a plane waves cut-off of 440 eV with a norm-conserving pseudopotential was implemented in the calculations. The structures were relaxed, including the unit cells, until the forces became smaller than 0.01 eV/Å and with self-consistent energy tolerances inferior to 5 × 10^−7^ eV/atom. For the monolayer case a vacuum of 21 Å between the layers was considered. Due to fact that the only symmetry operation present in the monolayer, few-layers, and bulk, is inversion symmetry, there is just one Raman active irreducible representation, i.e. *A*_g_. Thus, in Fig. 1e we have labeled the Raman peaks in a sequence from low to high frequencies as *A*_g_ with increasing exponent number. In the Supplementary Information, we included a table, i.e. Table S1, which provides the calculated phonon frequencies for all the bulk and monolayer Raman modes.

**Figure 2:**
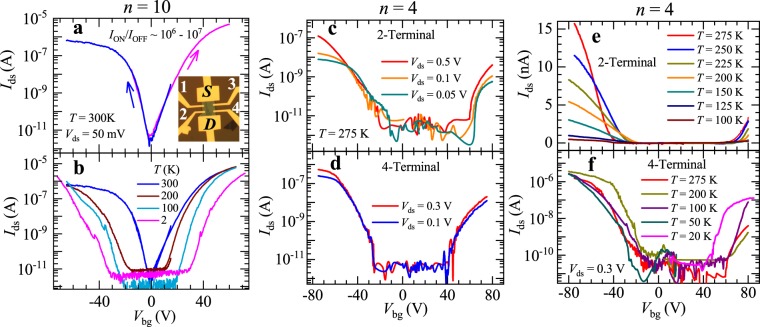
(**a)** Drain to source current *I*_ds_ as a function of the gate-voltage *V*_bg_ for a ten layer thick ReSe_2_ crystal. Blue (magenta) markers depict decreasing (increasing) gate-voltage sweeps. Notice the near absence of hysteresis. Both traces were acquired at room temperature under a bias voltage of *V*_ds_ = 50 mV. Inset: picture of the FET indicating the configuration of contacts. Source (S) and drain (D) contacts were used for two terminal measurements. The channel length and width of the device was 11.6 μm and 10.9 μm respectively. (**b**) *I*_ds_ as a function of *V*_bg_ for the same sample and for several temperatures ranging from *T* = 300 K to 2 K. Notice the progressive emergence of a threshold gate-voltage which increases upon decreasing *T*. (**c**) Drain to source current *I*_ds_ for a *n* = 4 sample as a function of the back-gate voltage *V*_bg_ in a semi-logarithmic scale for several values of the bias voltage *V*_ds,_ measured at *T* = 275 K through a two-terminal configuration. (**d**) Same as in (**c**) but measured *via* a four-terminal configuration. (**e**) *I*_ds_ as a function of *V*_bg_ for several temperatures measured via a two-terminal configuration in a linear scale. (**f**) Same as in (**e**) but in a logarithmic scale and measured through a four-terminal configuration. For both panels (e) and (f) a bias voltage *V*_ds_ = 0.3 V was used.

Figure 2 presents the overall electrical response of two field-effect transistors (FETs) built from exfoliated flakes composed of 4 and 10 layers, respectively. We observed a large variability in the response of the characterized FETs, with thicker crystals displaying considerably larger field-effect mobilities and smaller hysteresis and threshold gate-voltages relative to thinner ones as illustrated by both examples in Fig. 2 (see also Figs S1 and S2 in Supplementary Information file for electrical data from other samples). The lower mobilities and poorer overall electrical performance of FETs built from thinner ReSe_2_ crystals have been widely observed in transition metal dichalcogenides^30–32^ and attributed to a more pronounced role for Coulomb scattering from the impurities at the interface with the SiO_2_ layer and particularly from the adsorbates accumulated at the top layer of the semiconducting channel^30–32^. In thicker crystals/flakes the top layers play the role of capping layers, protecting the middle layers that carry most of the electrical current, from oxidation and adsorbates, while the bottom layers can partially screen the spurious charges at the interface. Simulations have also shown that carrier mobilities peak for samples having approximately 10 layers^30^. Figure 2a displays the drain to source current *I*_ds_, extracted under a bias voltage *V*_ds_ = 50 mV, as a function of gate voltage *V*_bg_ for a FET based on an *n* = 10 layers crystal. One observes i) the near absence of hysteresis and ii) a threshold gate-voltage *V*_th_ $\cong$ ±40 V beyond which conduction occurs at room temperature. Notice also the ambipolar behavior, or electron and hole-conduction, with ON to OFF current ratios of nearly ~10^7^ for electrons and of ~10^6^ for holes, albeit with poor subthreshold swings of typically ~3.5 V per decade. Previously ambipolarity was reported only for black phosphorus^24^, for MoSe_2_^33^ and for α-MoTe_2_^34^. Hence, ReSe_2_ becomes the fourth 2D material to display ambipolarity in absence of ionic liquid gating or dielectric, heterostructure or contact engineering, thus displaying a potential for applications in complementary logic electronics.

Figure 2b shows *I*_ds_ as a function of *V*_bg_ for the same sample but for several temperatures *T*. As *T* is lowered, one needs to reach progressively higher threshold gate voltages *V*^t^_bg_ to observe carrier conduction. We have observed this increase in *V*^t^_bg_ in most of the TMDs we have measured, ascribing it to a combination of factors, such as disorder-induced localization at the interface with the SiO_2_ layer, and the role of the Schottky barriers at the electrical contacts^35^. Figure 2c,d illustrate a comparison between 2- and 4-terminal measurements performed in a FET based on a ReSe_2_ crystal with *n* = 4 layers. Here, 2-terminal measurements indicate that current flows through the source and drain contacts which are also used for sensing the voltage. The overall response of this FET is noticeably inferior with respect to the *n* = 10 one: under the same bias voltage *V*_ds_ = 50 mV one extracts nearly 100 times less current leading to ON to OFF ratios reaching only 10^4^ and 10^3^ for holes and electrons, respectively. The rather large *V*^t^_bg_ of ~+40 and ~−30 V at *T* = 275 K for the *n* $\cong$ 4 sample suggests that this sample is considerably more disordered than the *n* $\cong$10 layers one: a large fraction of the initially accumulated carriers become trapped by defects and spurious charges in the material and at the interface. Figure 2e,f display *I*_ds_ as a function of *V*_bg_ for measurements based on 2- and 4-terminal configurations respectively, at several temperatures. Notice how the *V*^t^_bg_ are nearly *T*-independent, supporting the notion that they are associated with a constant number of defects in the material and/or with spurious charges at the interface.

**Figure 3:**
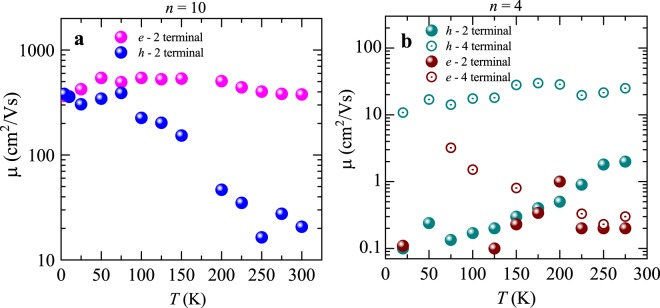
Electron- and hole- mobilities as a function of the temperature as extracted from the MOSFET transconductance formula for a (**a**) *n* = 10 and a (**b**) 4-layers thick sample. In (**a**) magenta and blue markers depict electron- and hole-mobilities respectively, as extracted from a two-terminal configuration under *V*_ds_ = 50 mV. In (**b**) dark cyan and brown markers depict hole- and electron mobilities, respectively. Solid and open circles indicate mobilities extracted from two- and four-terminal configurations, respectively. The drain to source voltage applied to the 4 layer sample was *V*_ds_ = 0.3 V.

Figure 3 displays the field-effect mobilities extracted from both samples (*n* = 10 and *n* = 4 layers) as a function of the temperature *T*, when using the conventional MOSFET transconductance formula, i.e. μ_FE_ = *c*_g_^−1^ *d*σ/*dV*_bg_, where σ = *j*_ds_/*E*_ds_ is the conductivity and *c*_g_ = *e*_r_*e*_0_/*d* = 12.116 × 10^−9^ F/cm^2^ is the gate capacitance (where *d* = 285 nm is the thickness of the SiO_2_ layer). For the thicker *n* = 10 sample, we measured the FET response only via the 2-terminal configuration. For the thinner *n* = 4 sample, we measured the mobility using the 2- as well as the 4-terminal method, since the first one yielded unusually small mobilites when compared to those of the *n* $\cong$ 10 samples. Our intention was to verify if this difference would be attributable to worse electrical contacts. Remarkably, for the *n* = 10 sample, whose data is shown in Fig. 3a, we observed nearly temperature independent electron mobilities with values around ~400 cm^2^/Vs. Meanwhile the hole mobilities varied significantly, increasing by more than one order of magnitude upon cooling, that is from ~20 cm^2^/Vs at room temperature to ~400 cm^2^/Vs at 75 K. Figure 3b displays the 2- as well as the 4-terminal electron and hole mobilities for *n* = 4 sample as a function of the temperature. Solid green and solid maroon dots depict hole and electron mobilities measured through a 2-terminal configuration. Open green and maroon dots depict hole and electron mobilities measured in a 4-terminal configuration, respectively. In the whole range of temperatures the mobilities of the *n* = 4 sample were considerably smaller than those of the *n* = 10 one, which displayed two-terminal hole mobilities on the order of just 1 cm^2^/Vs and electron mobilities one order of magnitude smaller. These values for the 2-terminal mobility of the thinner sample were very similar to those reported by Zhang *et al*.^36^ but are considerably higher than those reported for ambipolar α-(MoTe)_2_^34^.

The mobility values for the *n* = 10 sample were considerably higher than those previously reported for multilayered samples, which have been found to display electron-doped-like responses with two-terminal field-effect mobilities ranging from only ~1 to 10 cm^2^/Vs^1,12,36–38^. In Figs S3, S4, and S5 (see Supplementary Information) we included data from a second multi-layered sample, i.e. *n* = 8–9 layers, which also displayed field-effect electron mobilities in excess of 10^2^ cm^2^/Vs, with considerably smaller threshold gate-voltages relative to the thinner crystals. This indicates that thicker crystals display higher mobilities and that this behavior is not confined to the sample shown here. In thinner samples, charge conduction tends to be dominated by higher contact resistances when measured in a 2-terminal configuration. Therefore, in order to extract the nearly intrinsic mobility of the *n* = 4 sample we re-measured it through a 4-terminal configuration. From these measurements, we obtained hole-mobilities of ~10 cm^2^/Vs and an order of magnitude smaller electron mobilities at room temperature. This value for the 4-terminal hole-mobility is similar to the one reported by Zhang *et al*.^1^ on the same material after transferring onto h-BN substrates. In contrast, our electron mobilities are similar to their values extracted from ReSe_2_ FETs on SiO_2_. Unsurprisingly, this indicates that the substrates, i.e. their roughness, presence of dangling bonds and of trapped charges affect the mobilities of thin ReSe_2_ samples. Impurities should play a more predominant role in thinner crystals. The impurities to which we refer to are those located at the interface with the SiO_2_ layer as well as adsorbates on top of the channel resulting from air exposure during the fabrication process. Our observations would imply that previous reports underestimated the intrinsic performance of this compound. We discarded degradation under ambient conditions after evaluating the time dependence of the Raman signal, i.e. amplitude and width at half maximum of several of the observed Raman peaks as a function of time. We did not detect any deterioration over a time scale of a few days indicating that the previously discussed scattering mechanisms as well as the Schottky barriers at the electrical contacts are likely to be the main factors limiting the performance of ReSe_2_ field-effect transistors. Notice that the mobility μ^*h*^_FE_ of the holes in Fig. 3a increases considerably as *T* is lowered suggesting that it is phonon limited, or that phonons also play quite a relevant role at room temperature. In contrast, the electron and hole mobilities for the thin *n* = 4 layers sample show a quite different trend as a function of the temperature. The 2-terminal electron (μ^*e*^_2T_) and hole (μ^*h*^_2T_) mobilites decrease as a function of the temperature, indicating that the transport of charges is dominated by the Schottky barriers, or that the thermionic emission processes accross the contacts are suppressed at lower temperatures. This contrasting behavior also implies that the mobilities are sample dependent due in part to fluctuations in the quality of the contacts. The 4-terminal mobilities measured on the same device yields a hole mobility (μ^*h*^_4T_) that remains nearly constant at a value of ~20 cm^2^/Vs as a function of the temperature. In contrast, the electron mobility (μ^*e*^_4T_) increases from 0.3 cm^2^/Vs at 300 K to 3 cm^2^/Vs at 75 K. The previous report by Zhang *et al*.^1^ also found nearly constant mobilities as a function of the temperature when h-BN was used as the substrate.

**Figure 4:**
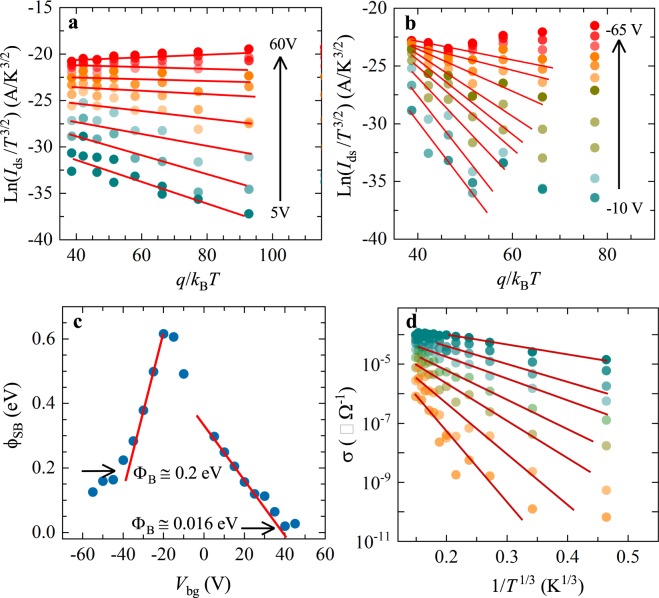
(**a)** Drain to source current *I*_ds_ normalized by a power of the temperature *T* as a function of the charge *q* = *e* for the *n* = 10 layers sample and for several positive values of the back gate voltage *V*_bg_. (**b**) Same as in (**a**) but for negative values of *V*_bg_. Both data sets in (a) and in (b) were measured under *V*_ds_ = 50 mV. In both panels red lines are linear fits from which we extracted the gate voltage dependence of the Schottky barrier ϕ_SB_ (*V*_bg_) between metallic contacts and the semiconducting channel. (**c**) ϕ_SB_ as a function of *V*_bg_, showing that in the limit of high gate voltages (flat band condition), the extracted Shottky barriers Φ_B_ were ~200 meV for holes and ~16 meV for electrons respectively. (**d**) Conductivity σ = *I*_ds_/*V*_ds_ *l*/*w*, where *l* and *w* are length and width of the semiconducting channel respectively, as a function of 1/*T*^1/3^ and for several gate voltages. Dark red lines are linear fits indicating that at lower temperatures the conductivity as a function of *T* can be described by the two-dimensional variable range hopping expression.

To evaluate the quality of the electrical contacts we performed two-terminal measurements in the *n* = 10 sample to evaluate *I*_ds_ as function of *V*_bg_ under a fixed *V*_ds_ = 50 mV at several temperatures, see Fig. 4. This evaluation is important since, as illustrated by Fig. S1 (see Supplementary Information), the *I*_ds_–*V*_ds_ characteristics are non-linear confirming a prominent role for the Schottky barriers at the level of the electrical contacts with a concomitant decrease in performance of the ReSe_2_-based FETs. The transport of electrical charges across a Schottky barrier, resulting from the mismatch between the band structure of the metal and that of the two-dimensional material, is usually described in terms of the two dimensional thermionic emission equation^39–42^:

1 $${I}_{{\rm{ds}}}=AA\ast {T}^{n}[-\frac{q{\varphi }_{{\rm{SB}}}}{{k}_{B}T}]$$

where *A* is contact area of junction, A* is the two-dimensional equivalent Richardson constant, *n* is an exponent acquiring a value of either 2 for a three dimensional semiconductor or of 3/2 for a two-dimensional one^42^, *q* = *e* is the electron charge, ϕ_SB_ is the Schottky barrier height, and *k*_B_ is the Boltzmann constant. In order to evaluate ϕ_SB_ or the effective Schottky barrier at the contacts, in the top panel of Fig. 4a and b we plot the drain-source current *I*_ds_ normalized by the power of the temperature *T*^3/2^ as a function of (*q = e*)/*k*_B_*T* as obtained under several values of *V*_bg_. Figure 4a corresponds to curves collected under *V*_bg_ > 0, while Fig. 4b corresponds to curves measured under *V*_bg_ < 0. Red lines in both panels are linear fits from which we extracted the value of ϕ_SB_ (*V*_bg_). Figure 4c displays the extracted values of ϕ_SB_ as a function of *V*_bg_. The Schottky barrier height Φ_B_ for electrons and holes were extracted from ϕ_SB_ at large absolute values of the gate voltage (flat band condition indicated here by deviations from linear fits) yielding values of ~0.016 and 0.2 eV, respectively. These values must be contrasted with the work function W = 5.6 eV and the band gap of Δ = 1.19 eV reported for ReSe_2_^12,43^. A Schottky barrier should be expected as the difference in energy between the work function of the deposited Cr contacts, or 4.5 eV, and the electron affinity *E*_EA_ $\cong$ (W − Δ/2) $\cong$ 5.005 eV of ReSe_2_, or Φ_B_ $\cong$ + 0.505 eV. This value implies that Cr should pin the Fermi level within the conduction band of ReSe_2_ thus explaining the rather small Φ_B_ ~ 0.015 eV extracted under positive gate voltages. The existence of a very small Schottky barrier could result from extrinsic factors like polymer residues resulting from the fabrication process. Remarkably, one also obtains a rather small Schottky barrier for holes of just Φ_B_ $\cong$ 0.2 eV, which is an unexpected result. Notice that a similar discrepancy was already observed by us for α-MoTe_2_^44^. Schottky barriers are likely the main factor limiting the hole-conduction in our ReSe_2_ FETs while their asymmetry would explain the larger electron mobilities.

**Figure 5:**
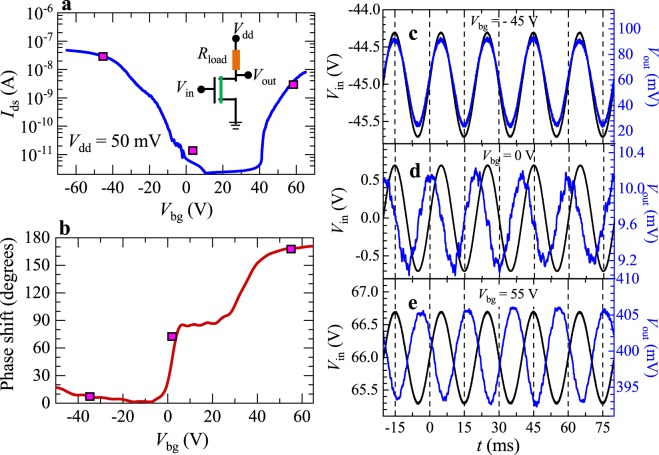
Phase-modulation based on a four-layer ambipolar ReSe_2_ FETs. (**a**) *I*_ds_ as a function of *V*_bg_ for a few-layer ReSe_2_ field effect-transistor at *T* = 275 K. This trace was acquired under a drain supply voltage *V*_dd_ = 50 mV. Inset depicts the scheme of measurements where *R*_load_ is a load resistor and *V*_dd_ is the bias voltage. *V*_in-ac,_ which is a superposition of DC and AC (~1.5 V) biases, was applied to the back-gate while *V*_out_ corresponds to the read-out voltage. Magenta squares indicate the DC back-gate voltages chosen to superimpose an oscillatory AC signal to extract the relative phase-shift between *V*_in_ and *V*_out_. (**b**) Relative phase shift as a function of *V*_bg_. By increasing *V*_bg_ from negative values we tuned the phase-shift to 90° and then to ~180°. This is clearly illustrated by panels (c,d) and (e) which display *V*_in_ (black traces) and *V*_out_ (blue traces) as a function of time *t* for various gate voltages.

Transistors displaying ambipolar behavior could be useful for applications in telecommunications since they could simplify circuit design or improve their performance for signal processing. For instance, we demonstrate that the ambipolarity of ReSe_2_ can be useful for the development of a phase shift modulator. For instance, Fig. 5 displays the response of a *n* = 4 ReSe_2_ based field-effect transistor, connected in series to a load resistor, upon the introduction of a sinusoidal modulation superimposed on its back-gate voltage, which we rename as the input-voltage, or *V*_in_ = *V*_bg_ + *V*_ac_ ($\cong$1.5 V). The readout oscillatory voltage *V*_out_ is collected at a point located between the load-resistor, in this case *R*_load_ = 100 kΩ, to which we apply a load voltage *V*_dd_ = 50 mV with respect to the ground, and the FET (see schematic of the circuit in Fig. 5a). Figure 5a also displays *I*_ds_ as a function of *V*_bg_ where we placed three magenta squares indicating the constant values of *V*_bg_ upon which oscillatory *V*_ac_ signals were superimposed while the corresponding *V*_out_ were collected. Figure 5b displays the phase shift of the *V*_out_ signal relative to *V*_in_ collected with a Lock-In amplifier as the gate voltage was swept from negative to positive values. Similarly to ambipolar α-MoTe_2_^34^, the relative phase between both signals was observed to shift from ~0° for *V*_bg_ < 0 V, to ~90° for 0 ≤ *V*_bg_ ≤ 20 V, and finally it nearly invertes to ~170° for *V*_bg_ > 40 V. These phase shifts are better illustrated by the raw oscillatory signals observed and collected with an oscilloscope as shown in Fig. 5c through 5e. When a negative *V*_bg_ was applied to the back gate, *I*_ds_ increased or decreased asynchronously with *V*_in_, and consequently the corresponding *V*_out_ also oscillated but in this case synchronously with *V*_in_. This configuration corresponds to the so-called common drain mode and is illustrated by Fig. 5c. In contrast, when a positive *V*_bg_ was applied, the corresponding *V*_out_ also oscillated although asynchronously, that is with a phase difference of nearly 180° with respect to *V*_in_, as shown in Fig. 5e (i.e. common-source mode). Remarkably, we observed a phase shift of ~π/2 for *V*_bg_ = 0 V which we attribute to the very high impedance of the FET for gate voltages inferior to the respective threshold gate voltages for conduction. The lack of a sizeable conductivity, or of a real component in the FET impedance, implies that its impedance is dominated by an imaginary component associated with, for example, the gate capacitance or capacitive and/or inductive couplings at the level of the contacts. Since the frequency is the rate of change of the phase, phase modulators can be used for frequency modulation (FM), and in fact they are employed in commercial FM transmitters. In addition to a phase shift modulator, the ambipolarity of ReSe_2_ can also be useful for the development of static voltage inverters, for example, by combining a ReSe_2_-based FET gated to display *p*-type behavior with another one gated to behave as *n*-type^2,45–47^. In supplementary Fig. S6 we included the phase-shift as a function of the gate voltage for a second sample having approximately 10 layers. Hence, this behavior is reproducible among samples having a different number of layers.

## Conclusions

In conclusion, given that the only symmetry operation present in the monolayer, few-layer and bulk ReSe_2_ is inversion symmetry, our Raman study coupled to density functional theory calculations indicate that their Raman spectra contain only modes belonging to the *A*_g_ irreducible representation. In addition and also in contrast to our previous studies on the isostructural ReS_2_ compound^18^, which was found to behave as an electron doped material, ReSe_2_ displays ambipolar behavior when contacted with Cr:Au electrodes. Relative to ReS_2_, we observed a considerably larger variability in the response of field-effect transistors fabricated from few layers of ReSe_2_ mechanically exfoliated onto SiO_2_. FETs based on ~10 layers of ReSe_2_ were observed to display up to one order of magnitude larger room temperature electron mobilities relative to FETs based on thinner flakes or on ReS_2_, with, remarkably, negligible threshold gate voltage for carrier conduction. This suggests that the material is intrinsically of high quality, or prone to a relative low density of defects. Given that Raman scattering as a function of time indicates that ReSe_2_ is rather stable under ambient conditions, the relatively poor performance observed in FETs fabricated from samples composed of just 3 to 4 layers is attributable to a poorer quality of the electrical contacts and to a more prominent role for impurity scattering from interfacial charges and adsorbates on the top layer. For instance, the exposure of the contact area to electron irradiation during the fabrication process is known to locally damage the material, for example, by inducing Se vacancies on its surface^18,48^. But as discussed in ref.^49^. Se vacancies can induce a large amount of interfacial states within the band gap leading, according to the DFT calculations, to nearly complete Fermi level pinning and possibly to larger Schottky barriers. Radiation induced defects should be particularly detrimental to monolayers, with their role weakening as the surface to volume ratio decreases or as the sample thickness increases. This would contribute to explain the superior performance observed by us on FETs based on 8–10 layers when compared to those composed of 3–4 layers. It would also contribute to explain the relatively low mobilities previously reported by other groups for this compound^1,12^.

Although our results point to considerably higher mobilities for the samples composed of *n* = 10 layers, one should take this observation with a grain of salt. For example, through a combination of measurements and simulations Das and Appenzeller concluded that four-terminal measurements would not be able to extract the intrinsic mobility of layered transition metal dichalcogenides given that both carrier concentration and mobility become spatially dependent^33^. In addition, one could also argue that we have not etched our crystals in a Hall bar geometry hence the metallic contacts deposited on the channel could affect its properties yielding incorrect values for its intrinsic mobility. However, we obtain comparable values for the 2- and the 4-terminal mobilities extracted for the *n* = 4 samples as well as similar values for the 2- and 4- terminal mobilities for the *n = *10 samples below *T* ~100 K. These observations, and their reproducibility in multiple samples with different geometries, strongly suggest that the higher mobilities for the *n* = 10 samples are intrinsic and do not a result from an artifact associated with the geometry or the position of the contacts.

The ambipolarity of ReSe_2_, when contrasted to the electron-doped behavior of ReS_2_, bears resemblance with the 2*H*-phase compounds MoSe_2_ and MoS_2_, where the former was reported by us as being ambipolar^50^ while the second is known for behaving as electron doped. In TMDs, the nature of the carrier conduction, i.e. electron- or hole-like, is usually attributed to Fermi level pinning associated with the Schottky barriers around the metallic contacts^51^. However, it seems difficult to reconcile this scenario with the differences in crystallographic and electronic structures between all of these compounds. Instead, it suggests that the electron character of MoS_2_ and ReS_2_ is intrinsically associated with the density of sulphur vacancies^52^. In any case, as we showed here, the ambipolarity of TMDs like ReSe_2_, allows one to produce quite simple logic elements having, for example, the ability to tune the phase of an incoming oscillatory signal towards 90° or 180° with the application of a single input voltage. It is therefore clear that these compounds have a remarkable potential for flexible logic applications. The current challenge is to understand and control the parameters limiting their performance, such as material quality, passivation, and Schottky barriers, in order to engineer commercial applications based on transition metal dichalcogenides.

## Materials and Methods

### Crystal synthesis

ReSe_2_ single crystals were synthesized through a chemical vapor transport (CVT) technique using either iodine or excess Se as the transport agent. Multi-layered flakes of ReSe_2_ were exfoliated from these single crystals using the micromechanical cleavage technique and transferred onto *p*-doped Si wafers covered with a 285 nm thick layer of SiO_2_.

### Characterization

Atomic force microscopy (AFM) imaging was performed using the Asylum Research MFP-3D* AFM. Raman spectra were acquired under ambient conditions using a micro-Raman spectrometer (Renishaw inVia micro-Raman). A grating of 1800 lines/mm was used in the backscattering geometry, and a 100 × objective lens was used to focus a laser spot size of ~1 μm onto the sample. The laser wavelength used to excite the samples was 514.5 nm (2.41 eV) from an Ar-Kr laser with a power around 0.1 mW to avoid any possible damage to the sample. Each Raman spectrum was measured with a 10 second accumulation time. Energy dispersive spectroscopy, to verify the stoichiometry, was performed through field-emission scanning electron microscopy (Zeiss 1540 XB).

### Trasmission electron microscopy

Sub-Angstrom aberration corrected transmission electron microscopy was performed with a JEM-ARM200cF microscope.

### Device fabrication

ReSe_2_ crystals were mechanically exfoliated and then transferred onto a clean 285 nm thick SiO_2_ layer. For making the electrical contacts 50 nm of Au was deposited onto a 5 nm layer of Cr via e-beam evaporation. Contacts were patterned using standard e-beam lithography techniques. After gold deposition, we proceeded with PMMA lift off in acetone. The devices were annealed at 300 °C for ~3 h in forming gas, followed by high vacuum annealing for 24 hours at 130 °C. Immediately after vacuum annealing, the devices were coated with a ~20 nm thick Cytop^TM^ (amorphous fluoropolymer) layer to prevent air exposure. Electrical characterization was performed by using a combination of a dual channel sourcemeters, Keithley 2400, 2612 A and 2635 coupled to a Quantum Design Physical Property Measurement System.

## Electronic supplementary material

Supplementary information

## Data Availability

The datasets generated and analyzed during the current study are available from the corresponding author on reasonable request. A part of these data are included in this published article as Supplementary Information file.
