# Supplementary material for: Phase Modulators Based on High Mobility Ambipolar ReSe2 Field-Effect Transistors
Source: Sci Rep. 2018 Aug 24;8:12745. doi: 10.1038/s41598-018-30969-7 (PMC6109127; doi:10.1038/s41598-018-30969-7)
Supplement: Supplementary file 1 — Supplementary information [file 41598_2018_30969_MOESM1_ESM.pdf]

Supplementary Information

Phase Modulators Based on High Mobility Ambipolar $ReSe_2$ Field-Effect Transistors

Nihar R. Pradhan[1,2], Carlos Garcia[2,3], Bridget Isenberg[2,4], Daniel Rhodes[2,3], Simin Feng[5], Shahriar Memaran[2,3], Yan Xin[2], Amber McCreary[6], Angela R. Hight Walker[6], Aldo Raeliarijaona[7], Humberto Terrones[7], Mauricio Terrones[5,8,9,10], Stephen McGill[2], & Luis Balicas[2]

[1]Department of Chemistry, Physics and Atmospheric Sciences, Jackson State University, Jackson, MS 39217, USA. [2]National High Magnetic Field Laboratory, Florida State University, Tallahassee, FL 32310, USA. [3]Department of Physics, Florida State University, Tallahassee, FL 32306, USA. [4]Lincoln High School, Tallahassee, FL 32311, USA. [5]Department of Physics and Center for 2-Dimensional and Layered Materials, The Pennsylvania State University, University Park, PA 16802, USA. [6]Engineering Physics Division, Physical Measurement Laboratory, NIST, Gaithersburg, Maryland 20899, USA. [7] Rensselaer Polytechnic Institute, Department of Physics, Applied Physics, and Astronomy, Troy, NY 12180 USA. [8]Department of Materials Science & Engineering, The Pennsylvania State University, University Park, PA 16802, USA. [9]Department of Chemistry, The Pennsylvania State University, University Park, PA 16802, USA. [10]Institute of Carbon Science and Technology, Faculty of Engineering, Shinshu University, Nagano 380-8553, Japan. Correspondence should be addressed either to N.R.P. (nihar.r.pradhan@jsums.edu) or to L. B. (balicas@magnet.fsu.edu)

Table S1. DFPT calculations (Density Functional Perturbation Theory) of the Raman Active modes of $ReSe_2$ (with asterisk).

Raman Active Mode	$ReSe_2$ Mono Layer Frequency (cm^{-1})	$ReSe_2$ bulk crystal Frequency (cm^{-1})
A_g^1	103.52	108.52
A_g^2	116.01	119.14
A_g^3	122.34	120.37
A_g^4	126.02	125.47
A_g^5	163.25	161.34
A_g^6	176.04	174.36
A_g^7	179.68	177.54
A_g^8	183.04	182.05
A_g^9	195.44	193.98
A_g^{10}	198.54	197.47
A_g^{11}	206.94	207.78
A_g^{12}	220.43	218.51
A_g^{13}	235.29	234.93
A_g^{14}	242.97	240.74
A_g^{15}	252.12	249.24
A_g^{16}	266.39	263.15
A_g^{17}	287.97	285.00
A_g^{18}	298.53	295.40

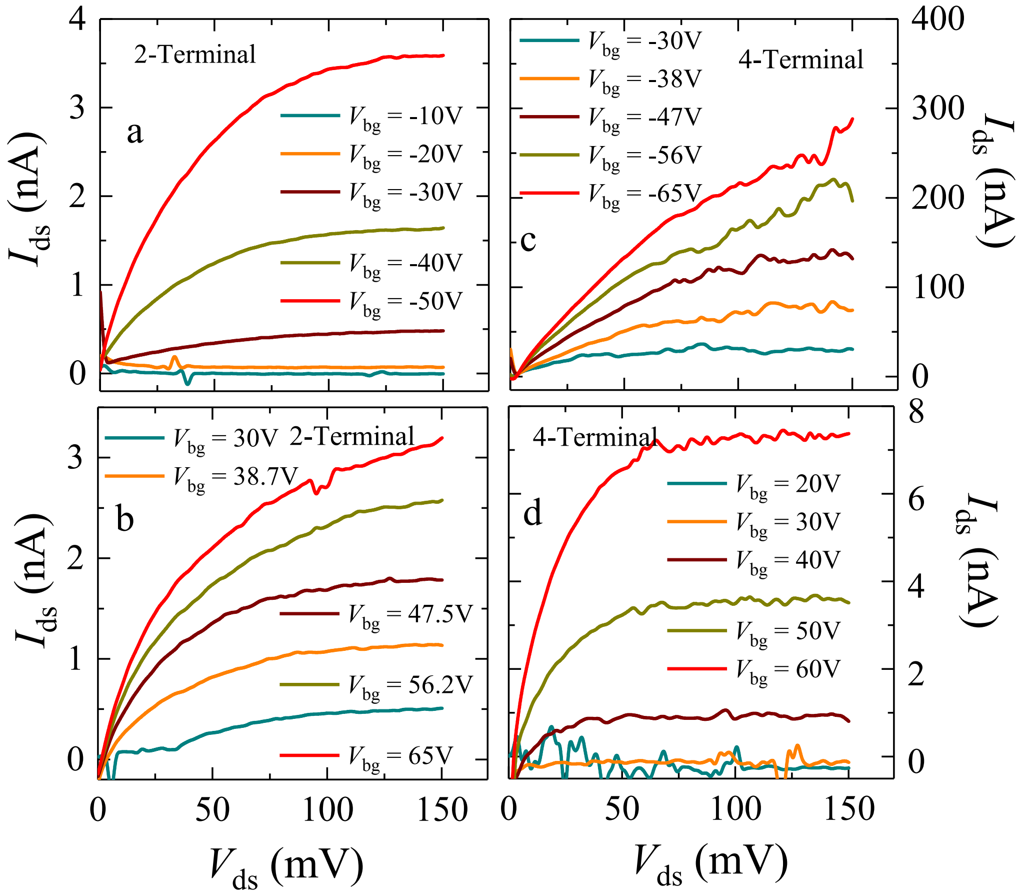

Figure S1. Drain to source current I_{ds} as a function of the bias voltage V_{ds} for a 3-4 layers $ReSe_2$ FET device. (**a**) and (**b**) I_{ds} as a function of V_{ds} measured with 2- and a 4-terminal configuration respectively, and for several negative values of the applied gate voltages V_{bg}. (**c**) and (**d**) I_{ds} as a function of V_{ds} measured *via* 2- and 4-terminal configurations, and for several positive values of V_{bg}.

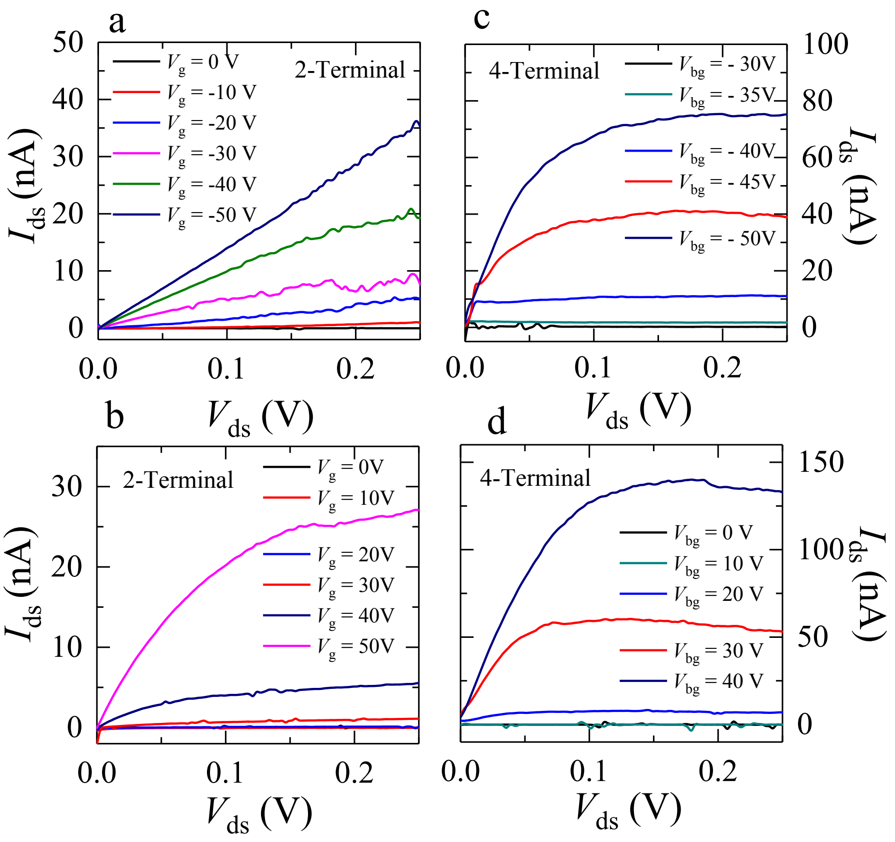

Figures S2. Drain to source current I_{ds} as a function of the bias voltage V_{ds} for a second thin (3-4 layers) $ReSe_2$ FET similar to the one discussed in the main text. (**a**) and (**b**) I_{ds} as a function of V_{ds} measured with 2 terminal configurations at several negative and positive gate voltages, respectively. (**c**) and (**d**) I_{ds} as a function of V_{ds} for several negative and positive values of the gate voltage measured with a 4-terminal configuration.

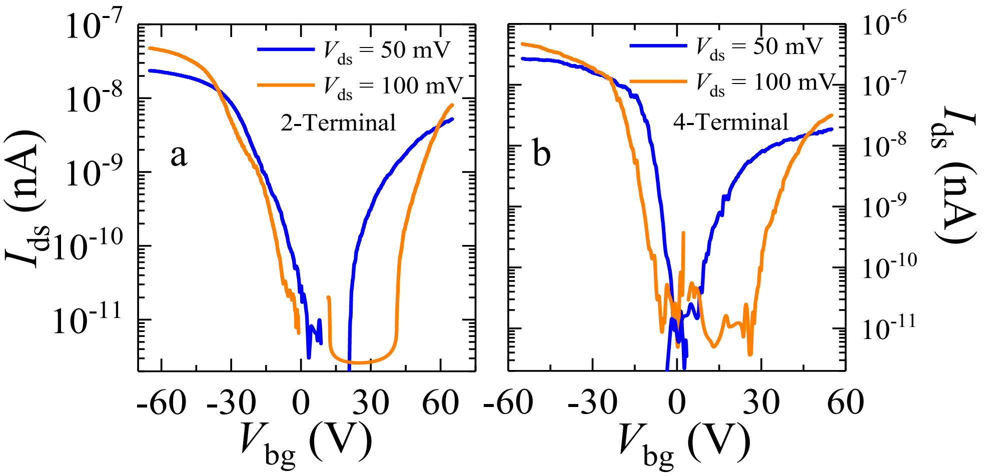

Figures S3. (**a**) and (**b**) Semi-logarthmic plot of I_{ds} as a function of V_{bg} for the device characterized in Figure S2 which was measured at room temperature using a 2-terminal and a 4-terminal configuration, respectively. Both configurations indicate ambipolar response characterized by a higher ON/OFF current ratio for holes with respect to electrons.

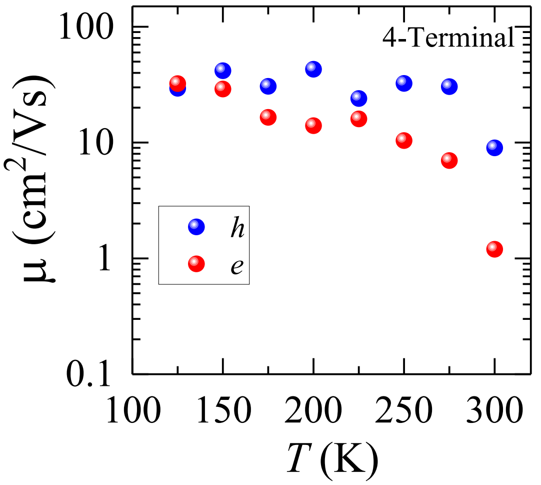

Figures S4. Field-effect mobility extracted from the previously discussed 3-4 layer thick sample measured *via* a 4-terminal configuration. The room temperature mobilities for electron and holes are in the order of ~1 cm^2/Vs and 10 cm^2/Vs, respectively. The mobilities increse upon cooling down saturating at a value of 40-50 cm^2/Vs below 200 K.

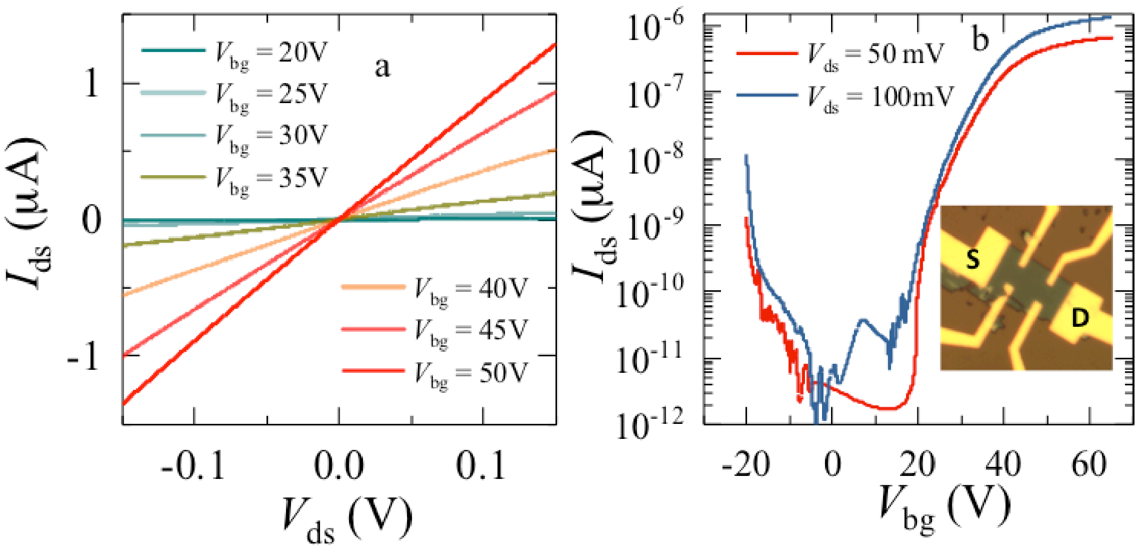

plates

Figure S5. Electrical characterization of a $ReSe_2$ based FET composed of 8 to 9 layers. (**a**) I_{ds} as a function of V_{ds} for several values of the applied gate voltage V_{bg}. Notice the nearly linear response at small bias. (**b**) I_{ds} as a function of V_{bg} displaying ambipolar response. For this sample one extracts an electron field-effect mobility of ~100 cm^2/Vs *via* a 2-terminal configuration. Inset shows the optical image of the $ReSe_2$ FET device of channel length L = 12.5 μm and width w = 6.7 μm.

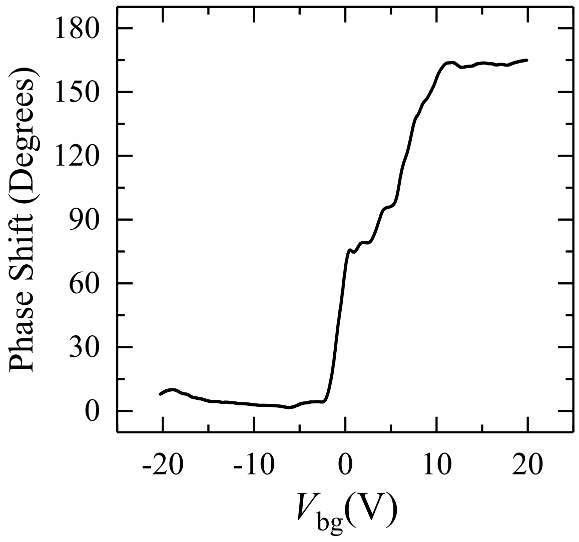

Figure S6. Relative phase-shift as a function of back gate voltage for a second, $n \cong 10$ layers, sample. Notice also that by increasing V_{bg} from negative values one is able to tune the phase to 90° and subsequently to $\sim 180^o$.
